# *Candida* blood stream infections observed between 2011 and 2016 in a large Italian University Hospital: A time-based retrospective analysis on epidemiology, biofilm production, antifungal agents consumption and drug-susceptibility

**DOI:** 10.1371/journal.pone.0224678

**Published:** 2019-11-07

**Authors:** Grazia Brunetti, Anna Sara Navazio, Alessandro Giuliani, Alessandra Giordano, Enrica Maria Proli, Guido Antonelli, Giammarco Raponi

**Affiliations:** 1 Department of Public Health and Infectious Diseases, Sapienza University of Rome, Rome, Italy; 2 Department of Molecular Medicine, Laboratory of Microbiology and Pasteur Institute-Cenci Bolognetti Foundation, Sapienza University of Rome, Rome, Italy; 3 Department of Environment and Health, Istituto Superiore di Sanità, Rome, Italy; 4 Microbiology and Virology Unit, Sapienza University Hospital Policlinico Umberto I, Rome, Italy; 5 Hospital Pharmacy, University Hospital Policlinico Umberto I, Rome, Italy; University of Palermo, ITALY

## Abstract

*Candida* bloodstream infection (BSI) represents a growing infective problem frequently associated to biofilm production due to the utilization of intravascular devices. *Candida* species distribution (n = 612 strains), their biofilm production and hospital antifungal drug consumption were evaluated in different wards of a tertiary care academic hospital in Italy during the years 2011–2016. In the considered time window, an increasing number of *Candida* BSI (p = 0.005) and of biofilm producing strains were observed (p<0.0001). Although *C*. *albicans* was the species more frequently isolated in BSI with a major biofilm production, an increased involvement of non-albicans species was reported, particularly of *C*. *parapsilosis* that displayed a high frequency in catheter infections, and lower biofilm production compared to *C*. *albicans*. Although trends of biofilm production were substantially stable in time, a decreasing biofilm production by *C*. *parapsilosis* in the Intensive Care Unit (ICU) was observed (p = 0.0041). Principal component analysis displayed a change in antifungal drugs consumption driven by two mutually independent temporal trends, i.e. voriconazole use in the general medicine wards initially, and fluconazole use mainly in the ICU; these factors explain 68.9% and 25.7% of total variance respectively. Moreover, a significant trend (p = 0.003) in fluconazole use during the whole time period considered emerged, particularly in the ICU (p = 0.017), but also in the general medicine wards (p = 0.03). These trends paralleled with significant increase MIC90 of fluconazole (p = 0.05), particularly for *C*. *parapsilosis* in the ICU (p = 0.04), with a general and significant decreased trend of the MIC90 values of caspofungin (p = 0.04), and with significant increased MIC50 values for amphotericin B (p = 0.01) over the study period. In conclusion, drug utilization in our hospital turned out to be a putative influencing factor on the ecology of the species, on the increase in time of the biofilm producing strains and on the *Candida* antifungal susceptibility profile, thus influencing clinical management.

## Introduction

Blood stream infections (BSI) caused by *Candida* have been reported as the fourth most common causes of nosocomial infections, both in Europe and in United States [1]. These infections have high morbidity and mortality rates, with significant influence also on the hospital costs (i.e. increased hospital length of stay, high costs for antifungal therapy) [2–5]. They are commonly related to several surgical and clinical procedures, such as parenteral nutrition, previous exposure to antibacterial therapy, chemotherapy, dialysis [1] as well as to the presence of intravascular devices (mainly CVC and urinary catheters) [6–8]. Catheterization and parenteral nutrition have been strongly associated to more serious and aggressive candidemia episodes, due to the onset of favorable conditions for the development of *Candida* biofilm. Biofilm formation is commonly associated with *Candida* BSI, since medical devices provide an optimal surface for *Candida* adherence, leading to an organized structure characterized by antifungal resistance, deeply influencing the management of the infection [9–11]. The real impact and possible correlation between biofilm formation and the clinical outcome of BSIs is still debated [2, 12–14]. Nowadays the management and therapies for *Candida* BSI rely on the patients’ underlying disease, on the immune status, on the patients’ risk factors for infection, and on the biofilm growth capacity that can influence the specific antifungal susceptibility profile. Fluconazole is still considered a first-line agent in *Candida* infections, but its efficacy is currently undermined by the growing emergence of azole-resistant *Candida* species [15–17]. Thus, current guidelines recommend echinocandins as first-line empirical treatment for most candidemia episodes and for biofilm associated infections, whether alone or in combination with catheter lock therapy [18]. Despite the outlined management strategies, the incidence of (both albicans and not-albicans) *Candida* BSI is increasing together with the emergence of biofilm producing and resistant species, [19–22] stressing the need for the prompt drug administration in order to improve the patients’ management. Purpose of this correlative observational study was to get a coarse grain estimate of the dimension and trend of the phenomenon. Therefore, we evaluated the yearly cumulative incidence of episodes in a tertiary care, University Hospital in Rome, Italy, analyzing the involved *Candida* species, the presence of intravascular devices, the distribution between hospital wards and the influence of biofilm formation in the *Candida* BSIs reported, along a six-year time period. Moreover, in order to investigate the possible changes in antifungal therapies administration, we examined the antifungal drug consumption in the different hospital wards during the considered time window. Changes in drugs’ MIC values were also evaluated so obtaining a general picture of the extension and temporal trends of *Candida* BSIs.

## Materials and methods

### Study design, strains and data collection

The study was conducted in the Laboratory for Clinical Microbiology of a 1.300 beds-multiple building, tertiary care, University Hospital (Policlinico Umberto I of Rome, Italy) with an average admission of 38.000 patients/year and lasted six years, from January 2011 till December 2016. Strains of *Candida spp*. were isolated from the blood and/or catheter tip cultures from patients hospitalized in the medical and surgical wards, as well as in the intensive care units (ICU). Neutropenic patients (i.e. those hospitalized in the onco-hematology unit) were not included in the study. The analyzed strains are relative to *Candida*-catheter-related bloodstream infections (CCRBSI) defined, according to standard microbiological criteria, as those cases when both catheter (mainly central venous catheter, CVC) tip and peripheral vein blood cultures yielded the growth of the same species of *Candida* with identical antifungal profile. Strains isolated from patients with *Candida* BSI, with either blood or catheter tip culture positive for *Candida* spp., not matching the aforementioned microbiological criteria, were also collected and defined as of non-CCRBSI origin. Patients’ data (ward of hospitalization, *Candida* species and number of isolation/year/1000 admission) were recorded and matched in an internal database. Records and information were anonymized by the laboratory staff members not involved in the study. Blood samples and CVCs were drawn by the healthcare professionals at the hospitalization site of the patients and sent to the clinical microbiology laboratory for routine analysis following standard diagnostic protocols, and therefore the authors did not collect clinical samples. Informed written patients’ consent was not required because of the observational nature of the study. The Ethical Committee of “Sapienza” University of Rome (Prot. 13/18) approved the study.

### Microbiological analyses

Blood samples were drawn in Bactec Mycosis IC/F or Bactec Plus Aerobic Broth and incubated in an automated culture system Bactec 9420 (Becton-Dickinson, Inc., Sparks, MD). Alternatively, blood samples were drawn in Bact/Alert Aerobic Culture Broth and incubated in the automated Bact/Alert Virtuo System (Biomerieux Diagnostics, Marcy-l’Etoile, France) for a maximum of five days. Positive samples were inoculated on Sabouraud CAF agar (Liofilchem srl, Italy) and cultured for at least 24 hours at 37°C. Catheter tips were microbiologically examined within 15 min after removal, according to the quantitative culture method of Cleri et al. [23] with slight modifications [24, 25]. After incubation at 37°C for 24 h, any growth of *Candida* on solid media was identified, by MALDI-TOF technique (Bruker Daltonik GmbH, Bremen, Germany) performed in duplicate on a single colony, accepting score values ≥ 1.8 [26, 27]. For each isolated strain, the minimal inhibitory concentration (MIC) of antifungal drugs was determined by Sensititre Yeast One plates (ThermoFisher Scientific, Waltham, Massachusetts, US), following manufacturer’s instructions. *C*. *parapsilosis* ATCC 22019 and *C*. *krusei* ATCC 6258 were used as susceptibility control strains. MIC 50 and MIC 90 values, defined as the minimal antifungal concentration with inhibitory effect on the growth of 50% and 90% of the *Candida spp*. strains respectively, were also considered.

### Biofilm production

The capacity of *Candida* strains to produce metabolic active fungal biofilm was measured *in vitro* by means of XTT [2,3-bis (2-methoxy-4-nitro-5-sulfophenyl)-2H-tetrazolium-5-carboxanilide, Sigma-Aldrich s.r.l., Milan, Italy] reduction assay [28], as previously described [25]. Briefly, each *Candida* strain in the log-phase of growth (200 μl of 10^6^ cells/ml in RPMI 1640 medium added with MOPS and 2% glucose) was plated on flat-bottomed 96-well polystyrene microtiter plates (Costar, Cambridge, MA, USA) and incubated at 37°C for 48h. After incubation, the plates were washed three times in sterile PBS to remove planktonic cells, and each well was added with 100 μL of XTT-menadione solution (0.67 g/L XTT, 1 μM menadione, Sigma-Aldrich). The resulting color change was spectrophotometrically quantified at 490 nm with a microtiter plate reader (EL808, Bio-Tek Instruments Inc., Winooski, VT, USA). In separate wells, 200 μl of modified RPMI 1640 medium and 100 μl of a phenazine methosulfate solution (50 mg/ml in water) (Sigma Aldrich) were used as negative and positive colorimetric controls, respectively. A stable biofilm producing *Candida albicans* strain (*C*. *albicans* SA40, kind gift from dr. F. De Bernardis, National Health Institute of Rome) was used as biofilm production control [29]. The analysis was performed in triplicate. Results were expressed in terms of biofilm-index (BI), relative to the control biofilm producer strain of *C*. *albicans*, according to the formula: BI = (absorbance test strain)/[0.5 (absorbance *C*. *albicans* SA 40)] as previously described [25]. A BI ≥ 0.5 identified a biofilm producer strain.

### Antifungal therapy and drug utilization data

Antifungal (amphotericin B, anidulafungin, caspofungin, fluconazole, itraconazole, micafungin, posaconazole and voriconazole) drug consumption was expressed as the Defined Daily Dose (DDD), assumed as the average maintenance dose per day for a drug used for its main indication in adults [30]. For each drug, wards’ utilization data expressed as total DDD per year/1000 admission were considered.

### Statistical analysis

The yearly cumulative incidence of *Candida* BSI was estimated in terms of the ratio between the number of new cases reported in the wards and the total number of patients hospitalized in the same ward each year, and expressed as the number of episodes / 1000 admissions. The production of biofilm by the yeast isolates was expressed as the mean±SD of the BI value. Statistical analyses were performed using SAS/STAT software. Correlation analyses, in the case of continuous variables, were based upon Pearson correlation coefficient metrics in both direct and partial correlation mode, to decouple correlations of interest from eventual confounding factors so to sketch a reliable interpretation of the observed findings [31]. Principal Component Analysis (PCA) was instrumental to highlight the dynamics of change of drug profile use across different wards in time [32]. We adopted a chi-square correlation metrics when in presence of categorical variables while Analysis of Variance strategies (in both the forms of simple regression and general linear model) were adopted for inferential approach.

## Results

### Microbiological data analysis

**Fig 1:**
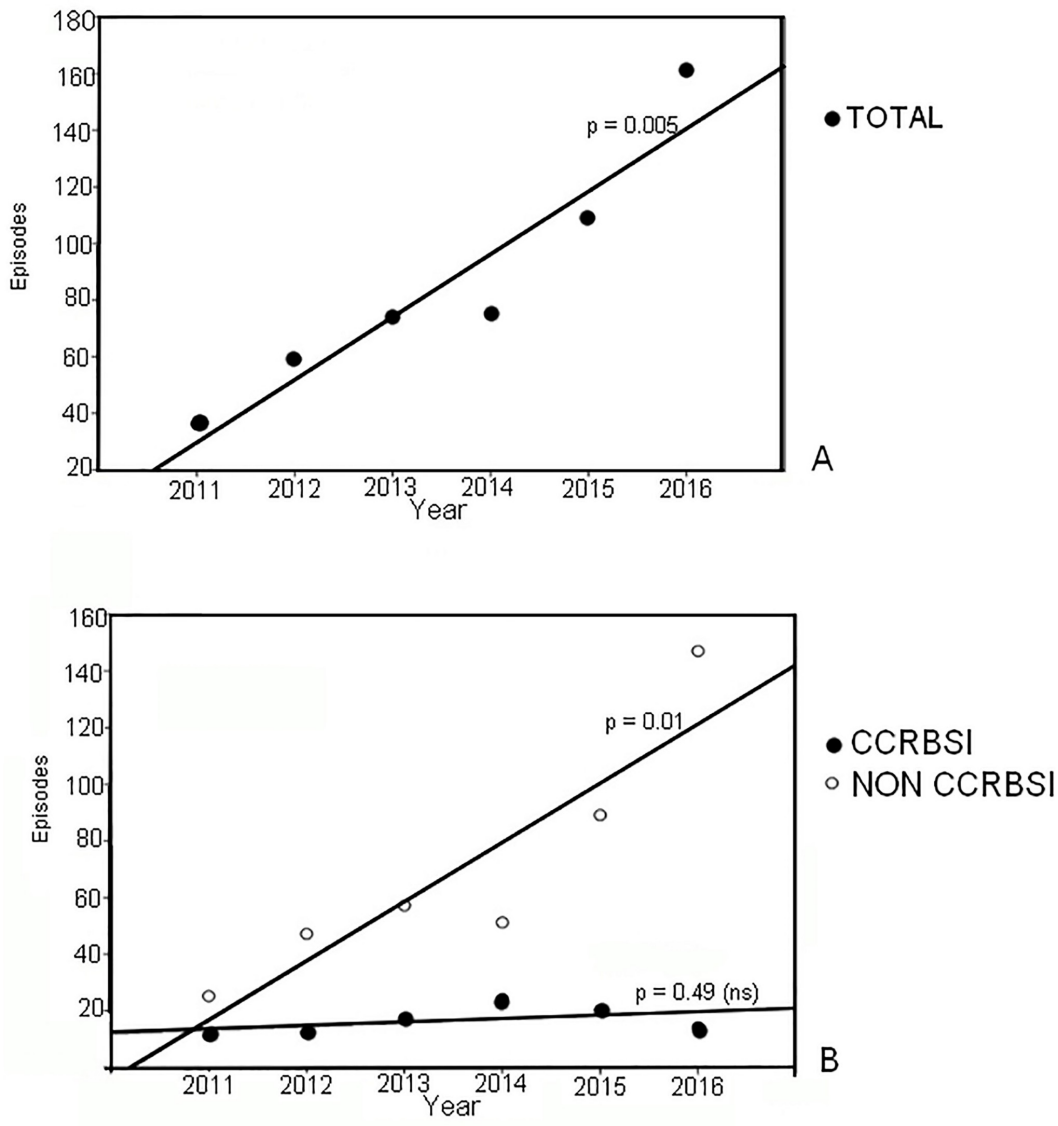
Yearly trend of total candidemia episodes (A), and of (CCRBSI) and non-*Candida*-catheter-related bloodstream infection (non-CCRBSI) (B).

A total of 514 episodes of *Candida* BSI were observed in the study period. The number of episodes increased between 2011 and 2016 in a statistically significant (Pearson’s test r = 0.944; p = 0.005) manner (top panel of Fig 1). When the episodes were partitioned into non-CCRBSI and CCRBSI, the overall frequency of non-CCRBSI episodes was higher than that of CCRBSI (67.97% vs. 32.03%, respectively). The non-CCRBSI episodes had the same trend of the total episodes (r = 0.90, p = 0.01). At odds with non-CCRBSI, CCRBSI episodes did not show any statistically significant increase in the studied time window (bottom panel of Fig 1). The estimated average yearly rate of increase was around 20 cases for total and non-CCRBSI episodes (22.17 and 20.85 respectively, Fig 1). A total number of 612 *Candida* strains were collected from the infective episodes in the considered period: *C*. *albicans* was the most frequently isolated species (43.63%), followed by *C*. *parapsilosis* (40.85%), *C*. *glabrata* (6.86%), and *C*. *tropicalis* (5.56%). *C*. *parapsilosis* accounted for the majority (72.5%) of the isolates among the not-albicans species. We partitioned the entire strains collection according to species, site of isolation, ward in which the episode was registered, and CCRBSI or non-CCRBSI episode (Table 1). The majority of the strains (65.20%) were isolated from blood samples, with a higher frequency of *C*. *albicans* (31.21%) followed by *C*. *parapsilosis* and *C*. *glabrata* (24.35% and 4.08%, respectively). Considering CVC samples, *C*. *parapsilosis* was the species more frequently isolated (16.50%), followed by *C*. *albicans* and *C*. *glabrata* (12.42% and 2.78% respectively). The above described species distribution was reported to be correlated with sites of isolation (Chi square p = 0.0324, Table 1). Considering the ward of hospitalization, the highest prevalence of *Candida* BSIs was detected in the ICU ward (31.5 episodes/1000 admission), while the wards of internal medicine and surgery were characterized by far lower rates (0.0026 and 0.0014 episodes/1000 admissions, respectively). The profiles of the species distribution were substantially invariant (Pearson r between 0.989 and 0.999) across the different wards so pointing to a common ‘ecology’ of the hospital. At any rate, however, *C*. *parapsilosis* was the most frequently detected species in the ICU followed by *C*. *albicans* (15.4 and 12.9 episodes/1000 admissions, respectively), which was instead the most often isolated species in the general medicine and surgery wards (0.0012 and 0.0006 episodes/1000 admissions, respectively). In the non-CCRBSI group, *C*. *albicans* was the species with the higher isolation rate, followed by *C*. *parapsilosis* and *C*. *glabrata*. Conversely, in the CCRBSI group *C*. *parapsilosis* was the species most often involved, followed by *C*. *albicans*, *C*. *glabrata* and *C*. *tropicalis*. Species distribution in the two groups (non-CCRBSI and CCRBSI) was significantly different (Chi Square test p = 0.0039).

**Table 1:** Distribution of *Candida* species. Number (percentage) of *Candida* species isolated in the study, according to isolation site, wards (ICU: Intensive Care Unit, INT. MED: Internal Medicine, SUR: Surgical wards), CCRBSI and non-CCRBSI. Other species included *C*. *gulliermondii*, *C*. *krusei*, *C*. *lusitaniae and C*. *palmiolephilia*.

Species detected	Number of strains [N(%)]	Isolation site [N(%)]		CCRBSI [N(%)]		CCRBSI/Ward [N/1000admission]		
		CVC	Blood	Yes	No	ICU	INT. MED	SUR
*Candida albicans*	267 (43.63)	76 (35.68)	191 (47.87)	67 (34.18)	200 (48.07)	12.9	0.0012	0.0006
*Candida parapsilosis*	250 (40.85)	101 (47.42)	149 (37.34)	101 (51.53)	149 (35.82)	15.4	0.0010	0.0005
*Candida glabrata*	42 (6.86)	17 (8)	25 (6.3)	12 (6.12)	30 (7.21)	2.1	0.0002	0.0001
*Candida tropicalis*	34 (5.56)	10 (4.7)	24 (6)	12 (6.12)	22 (5.29)	0.3	0.0002	0.0001
*Other species*	19 (3.10)	9 (4.2)	10 (2.4)	4 (2.05)	15 (3.61)	0.7	0.0001	0.00005
Total	612 (100)	213 (34.80)	399 (65.20)	196 (32.03)	416 (67.97)	31.5	0.0026	0.0014

### Biofilm production

**Fig 2:**
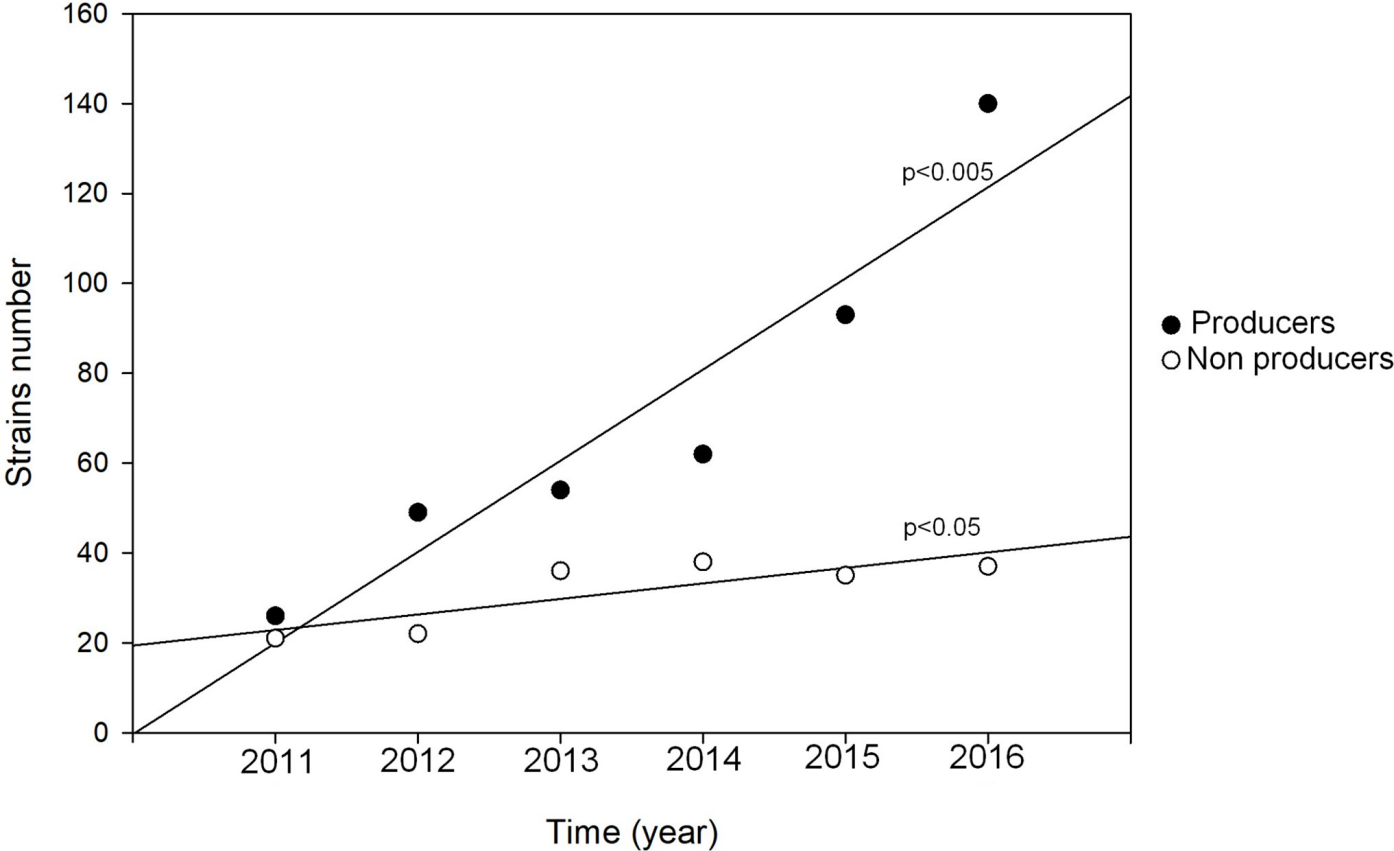
Time trend of biofilm producer and non-producer strains. Biofilm producer strains showed a much higher increase than non-producer strains during the considered time window pointing to a dramatic rise of producer/non-producer ratio (producer: r = 0.942 F = 31.25 p<0.005 estimated yearly increase 20.29 vs non-producer: r = 0.827 F = 8.64 p<0.05 estimated yearly increase 0.20).

To verify the capacity of biofilm production by *Candida* isolates, experiments were performed measuring the metabolic active fungal biofilm by means of XTT reduction assay. The results were expressed in terms of Biofilm Index (BI) and the isolated strains were divided into producers (BI ≥ 0.5) and non-producers (BI<0.5). Both producer and non-producer strains showed a statistically significant increase in the six-year period considered (Fig 2). However, the rate of increase of biofilm producer strains was ten-times higher than that of the non-producer so highlighting a neat change in the producer/non-producer ratio, with an estimated yearly increase of the number of producer strains 100 times higher than that of the non-producers (Fig 2). When the infectious episodes were coded according to the causative biofilm producing/non-producing strain, we observed that 69.28% of yeasts were producers and a statistically significant association between the isolated species and biofilm production capacity was observed (p<0.0001, Chi square test, Table 2). *C*. *albicans* and *C*. *tropicalis* were the species with the highest intrinsic biofilm production (Table 2). When passing from the binary yes/no production of biofilm in the different infective episodes to the actual amount of biofilm production (Table 2) we confirmed the among strains differences in biofilm production capacity by analysis of variance (p < 0.0001). Furthermore, we observed that the species more frequently represented among the producing strains were *C*. *albicans* and *C*. *parapsilosis* (246 and 130 producer strains, respectively).

**Table 2:** Biofilm production by the isolated yeasts. Strains were considered producers when the biofilm index (BI), obtained from the ratio between the absorbance of the considered strain / 0.5 absorbance of the control strain (*C*. *albicans* SA 40), was ≥ 0.5. Other species included *C*. *gulliermondii*, *C*. *krusei*, *C*. *lusitaniae and C*. *palmiolephilia*.

Detected species	Biofilm production			
	N° of producer strains (%)	Mean BI	N° of non-producer strains (%)	Mean BI
*Candida albicans*	246 (92.13)	2.47±1.25	21 (7.87)	0.287±0.141
*Candida parapsilosis*	130 (52)	1.573±0.811	120 (48)	0.218±0.147
*Candida glabrata*	8 (19)	0.731±0.189	34 (81)	0.237±0.112
*Candida tropicalis*	30 (88.24)	1.359±0.541	4 (11.76)	0.355±0.116
*Others*	10 (51)	0.884±0.378	9 (49)	0.260±0.105
Total	424 (69.28)	1.403±0.687	188 (30.72)	0.271±0.053

**Fig 3:**
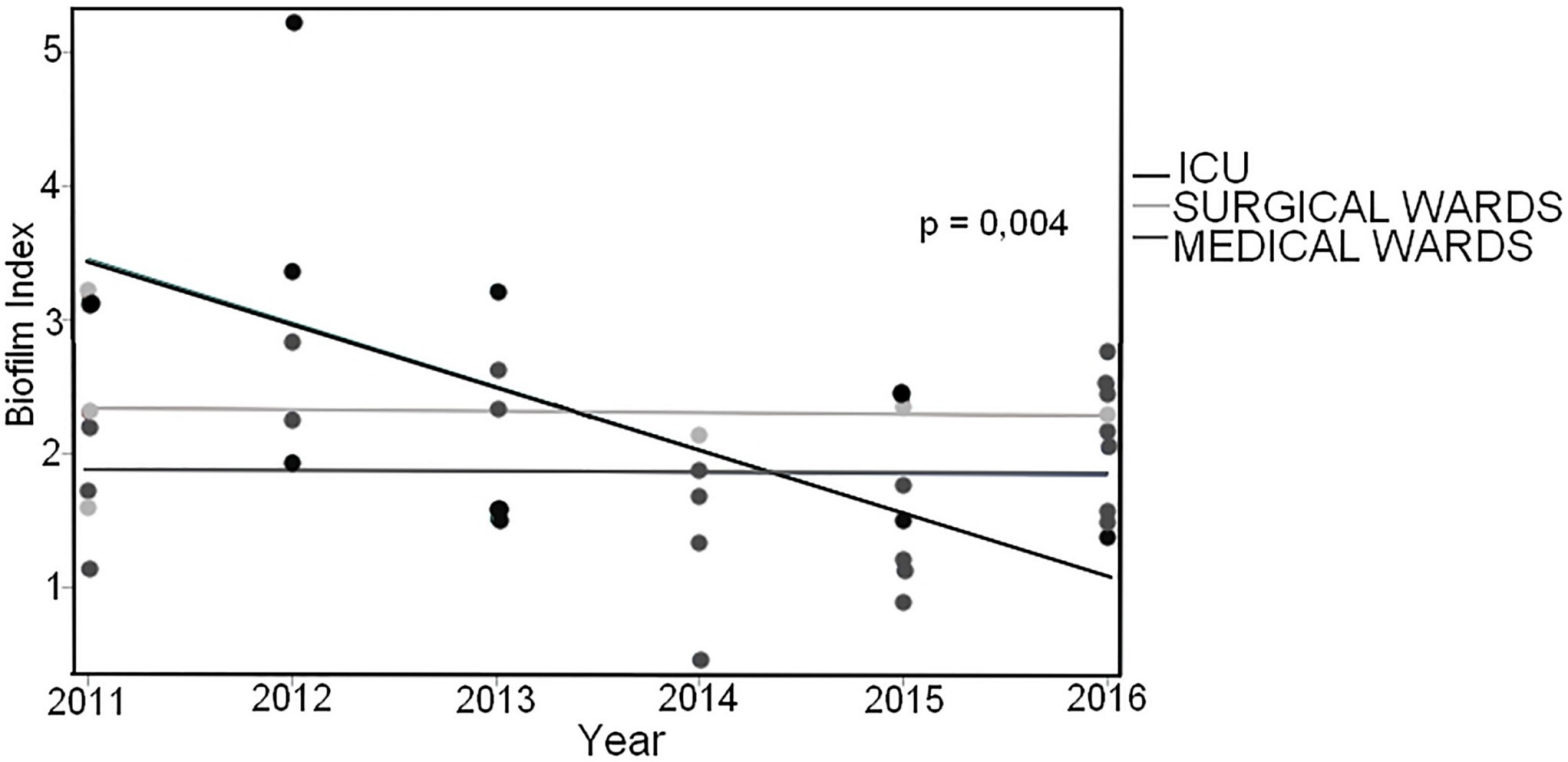
Reduced trends of the BI values produced by *C*. *parapsilosis* in the ICU compared to other wards. The figure has as X axis the time course, while dependent Y axis corresponds to biofilm index (BI) measured in a-dimensional units correspondent between the absorbance of the considered strain / 0.5 absorbance of the control strain (*C*. *albicans* SA 40). The statistical significance indication reported in the figure refers to the linear time trend of ICU.

When the series were stratified in CCRBSI and non-CCRBSI episodes, statistically significant (p = 0.0012) association emerged between CCRBSI and being a biofilm producing strain. Furthermore, significant (p = 0.0073) correlation between BI values and being a producer strain in the CCRBSI group was observed. In the CCRBSI, infectious episodes were more frequently sustained by *C*. *parapsilosis* characterized with a lower biofilm production capacity (mean BI = 1.528±0.69) as compared to *C*. *albicans* (mean BI = 2.157±0.84) that in turn was an efficient biofilm producer. Analyzing the trend of BI values in the different wards, we observed that the cumulative incidences were substantially stable in time, regardless of the species involved. Statistically significant correlations in time emerged in the CCRBSI group as for the BI values of *C*. *parapsilosis* isolated in the ICU (p = 0.0041). ICU displayed a significantly different behavior with respect to the other wards both as time trend (p = 0.0058) and as global incidence (p = 0.0042). A significant reduced trend of the BI values produced in the ICU by *C*. *parapsilosis* was observed in the study period as compared with that observed in the other wards (Fig 3, time*ward interaction effect p = 0.0007).

### Antifungal therapy and drug utilization data

**Fig 4:**
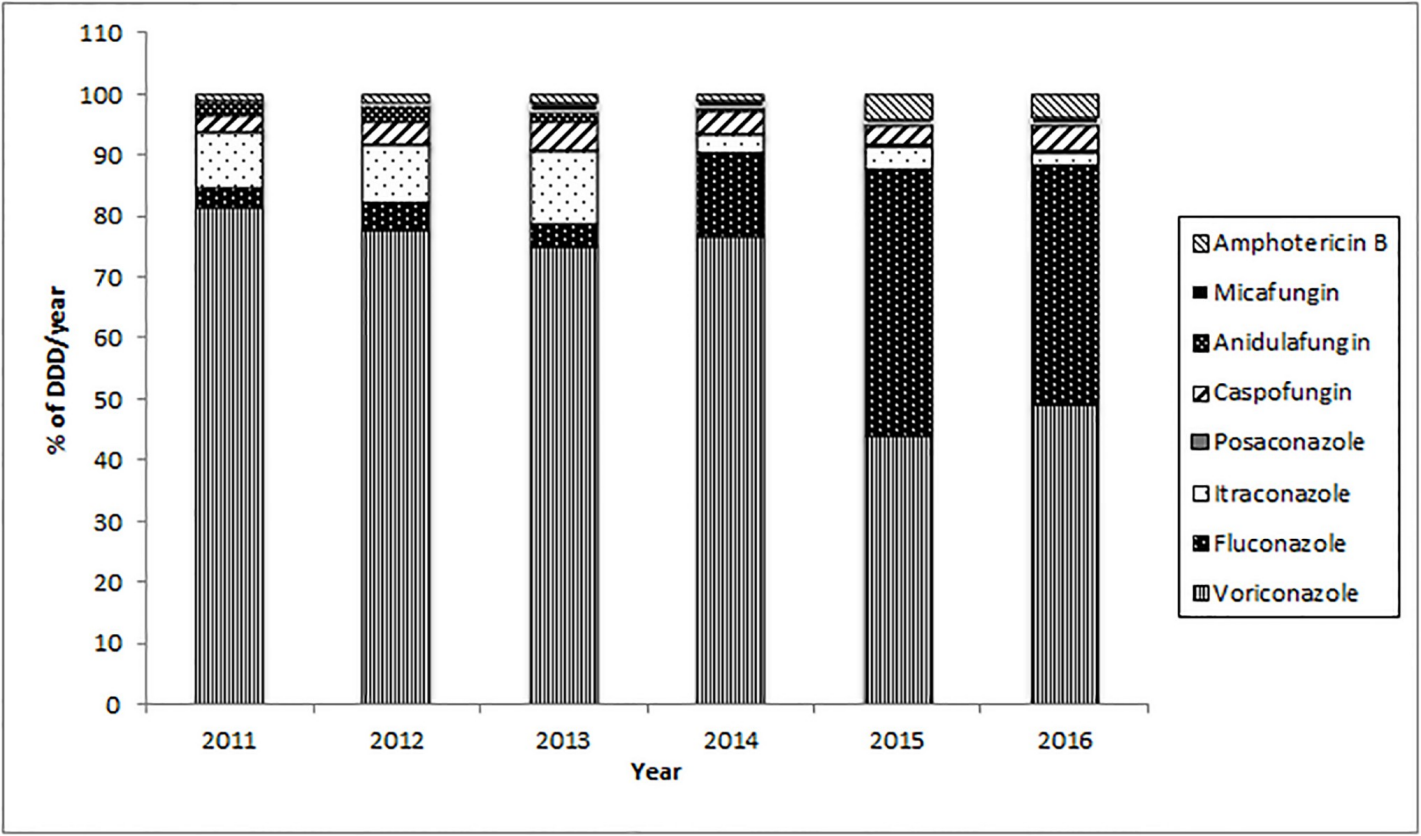
Normalized distribution (being 100 the total) relative to different years of the antifungal drugs (FLC, fluconazole; VRC, voriconazole; POS, posaconazole; ITC, itraconazole; CAS, caspofungin; AND, anidulafungin; MIC, micafungin; AMB, amphotericin B). The data are expressed as Defined Daily Dose (DDD) per year.

**Fig 5:**
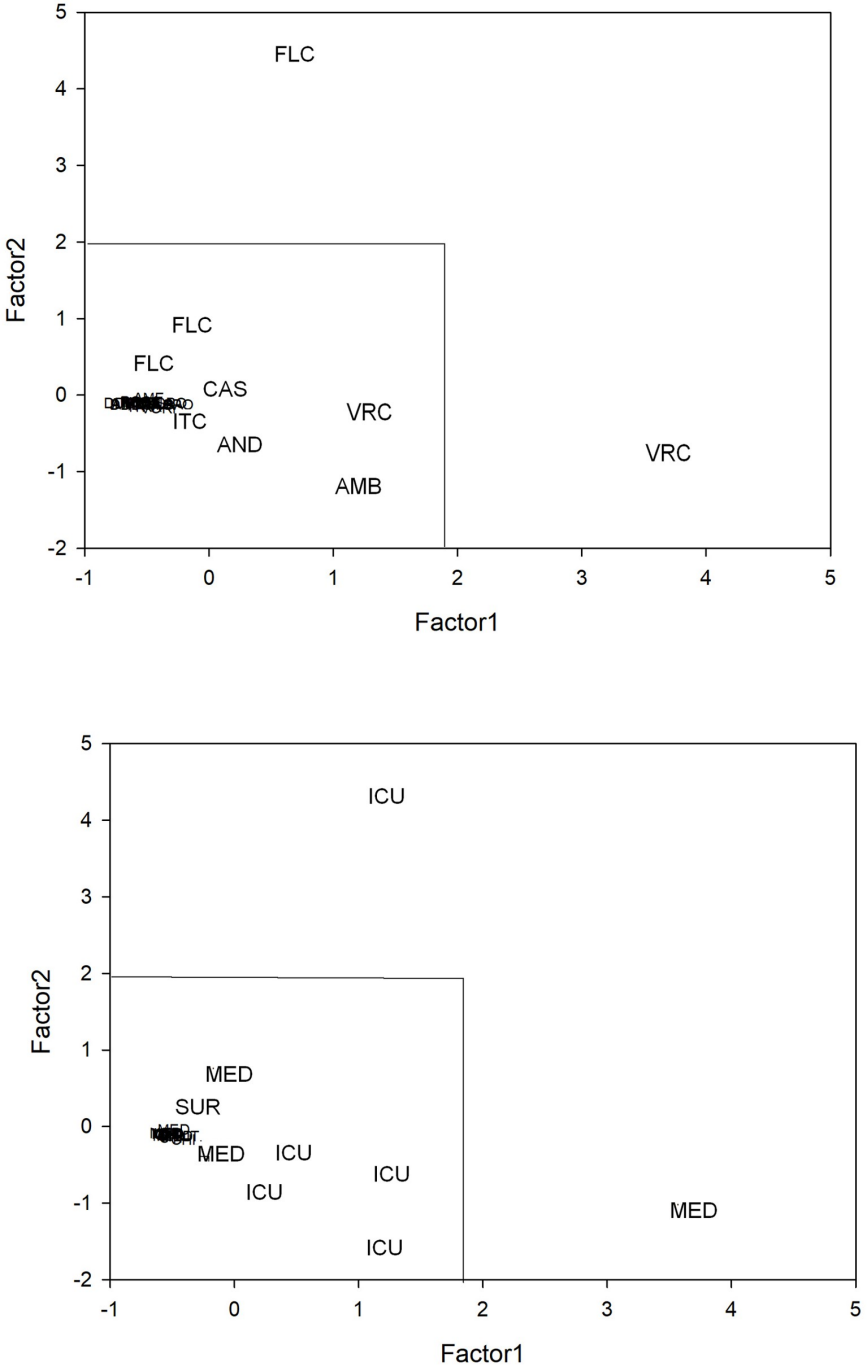
Bi-dimensional representation of the main drivers of the antifungal use change in time. The axes of the figure correspond to the principal component space spanned by the two main factors explaining approximately the 95% of system variance. The vector points in the top panel are expressed with antifungal drugs abbreviations, while in the bottom panel the same statistical units are defined by the correspondent ward. Given the factors (component) are computed as z-scores (zero mean and unit standard deviation) and are mutually independent by construction, the square at the bottom left part of the plot corresponds to the ‘within 95% confidence interval area’.

**Fig 6:**
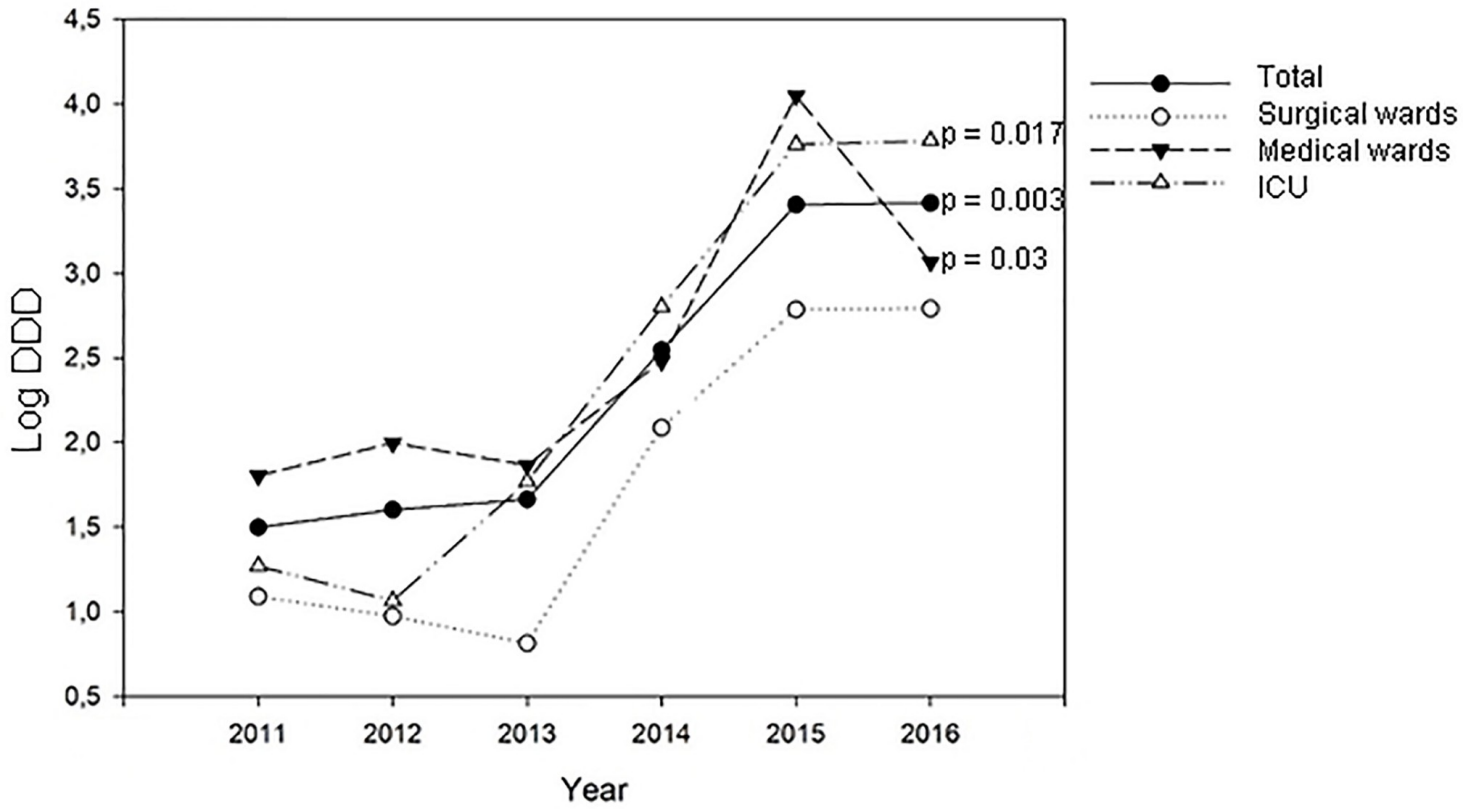
Trend in fluconazole use, in different wards, during the period considered in the study. The figure has as X axis the time course, while dependent Y axis corresponds to Defined Daily Dose expressed on a logarithmic basis.

To ascertain whether antifungal drug therapy could correlate with the number of *Candida* BSI episodes and with the variations of antimicotic susceptibility pattern in terms of MIC50 and MIC90, the drug consumption in the different wards was analyzed. Therefore, drug consumption expressed as DDDs concerning amphotericin B, anidulafungin, caspofungin, fluconazole, itraconazole, micafungin, posaconazole and voriconazole use was evaluated and expressed as the annual percentage of the total antifungal consumption (Fig 4). Principal Component Analysis (PCA) was applied to total DDD per year, i.e. to the data set having as rows the 24 (3X8) statistical units correspondent to the use of the eight antifungals in the three wards and as variables (columns) the different years. This allowed us to get a comprehensive picture of the temporal structure of fluctuations of the relative use of the different anti-fungal therapies [32]. PCA gave rise to a two component (factors) solution getting rid of the temporal dynamics of drug use profiles (cumulative explained variance = 94.6%). The two components corresponded to the mutually independent (and coexisting) dynamics of change along time (extracted factors are each other independent by construction): the loading pattern (correlation coefficients between original variables and components) is reported in Table 3. Factor1 and Factor2 explain 68.9% and 25.7% of total variance respectively, reflecting the variations of antifungal usage during the observed period with a very neat transition in year 2015. Looking at Table 3 it is evident how the ‘global trend’ correspondent to Factor1 (‘size component’) [33], starting from 2015, co-exists with a new dynamical structure (Factor2) collecting the higher proportion of 2015–2016 information (more elevated loading). This second component was substantially irrelevant in the preceding years (very low loadings with 2011–2014 years). Projecting the different statistical units (wards/antifungals) in the bi-dimensional plane spanned by the two factors, it was possible to identify the main drivers of the drug use change in time (Fig 5). Being the two factors linearly independent and having, by construction, zero mean and unit standard deviation, we identified a ‘bi-dimensional’ confidence square (limits -2 and 2, confidence of approximately 95%), collecting all antifungals (top panel) and the wards (bottom panel) that did not significantly depart from ‘common trends’. The points outside the intervals were significantly different from the general trend, i.e. they could be considered as ‘singular points’ driving the profile changes. In particular, the first factor mirrored voriconazole use in the general medicine wards, with almost constant administration during the first 4-year time period, while the second one showed a correspondence to fluconazole use in the ICU, with a strong increase during the last 2 years considered (Fig 5). In general, apart from anidulafungin and itraconazole, an increasing trend of drug consumption was observed consistent with the above-mentioned significant enhanced trend of *Candida* BSIs. Although the trend of consumption did not significantly change during the study period, the usage of amphotericin B and caspofungin in 2015 and 2016 was respectively seven and four times higher than the mean consumption observed in the years from 2011 to 2014. Intriguingly, a significant trend (p = 0.003) in fluconazole use during the whole time period considered emerged, particularly in the ICU (p = 0.017), but also in the general medicine wards (p = 0.03), with a clear tipping point correspondent to the year 2015 (Fig 6). Together with the analysis on the antifungal data consumption, MIC50 and MIC90 trends during the period considered were evaluated. For all the investigated species, a general increased trend in fluconazole MIC90 values was highlighted (r = 0.794, p = 0.05). This increase was particularly observed for MIC90 values of *C*. *parapsilosis*, in the ICU ward (r = 0.823, p = 0.04). On the other hand, a statistically significant (r = -0.83, p = 0.04) decrease in fluconazole MIC50 values was observed in medicine wards. Intriguingly, when considering only the biofilm producer strains, an increased trend in fluconazole MIC90 values was observed for both *C*. *parapsilosis* and *C*. *albicans* (r = 0.794, p = 0.05 and r = 0.92, p = 0.008 respectively). The cumulative data concerning caspofungin witnessed a general and significant decreased trend of the MIC90 values (r = -0.83, p = 0.04). However, the trend of the MIC50 values detected in medicine wards was significantly (r = 0.92, p = 0.01) increased. In particular, the trend of the MIC50 and of the MIC90 of caspofungin for *C*. *albicans* relative to the strains isolated from the medicine wards, showed significantly increased values (r = 0.828, p = 0.04 and r = 0.925, p = 0.01, respectively). Similarly, the trends of the MIC50 values for amphotericin B of both *C*. *albicans* and *C*. *parapsilosis* showed that the values increased significantly (r = 0.90, p = 0.01) over the study period.

**Table 3:** PCA analysis of DDD/year: Dynamics of change during time. The bolded values correspond to the variables most relevant for interpreting the latent factors (principal components) of the dynamics.

YEAR	FACTOR 1	FACTOR 2
2011	**0.927**	-0.335
2012	**0.885**	-0.373
2013	**0.908**	-0.259
2014	**0.954**	-0.087
2015	0.600	**0.789**
2016	0.631	**0.770**
Explained variance	68.9%	25.7%

## Discussion

The emergence of *Candida spp*. as one of the leading causes of BSI in our hospital has been witnessed by the statistically significant increase of the number of *Candida* BSI from 2011 to 2016 (p = 0.0005), confirming our previous observations [24]. These data paralleled those of other studies both at national and international level [21, 34–37]. The strain collection of this study revealed that between 2011 and 2016 the species more frequently observed was *C*. *albicans* (43.63%), followed by *C*. *parapsilosis* (40.85%), *C*. *glabrata* (6.86%), and *C*. *tropicalis* (5.56%). These data confirmed previous observations that *C*. *albicans* was the species more frequently involved in bloodstream infections [38–41]. Furthermore, an intriguing observation in our study was the high number of isolations of not-*C*. *albicans* strains, which caused 56.37% of the *Candida*-related episodes, thus pointing to an increased involvement of non-albicans species in the development of BSIs. In particular, *C*. *parapsilosis* accounted for more than 70% of the non-albicans species isolated in the infective episodes and was the species more frequently isolated from CVCs and in the CCRBSI group. Interestingly, considering the presence of intravascular devices (i.e. CVC) and dividing the episodes in catheter-related and not-related BSI, we observed that the frequency of non-CCRBSI episodes was higher than that of CCRBSI (67.97% vs 32.03%, respectively) and that the trend-in-time for this subgroup was statistically significant. Species distribution between CCRBSI and non-CCRBSI subgroups resulted significantly different (p = 0.0039) highlighting the presence of two different populations: one mainly composed by *C*. *albicans* strains, observed with high frequency in both CCRBS and non-CCRBS infections; the second headed by *C*. *parapsilosis* that included the species sustaining the majority of the CVC-related infections. Taking into account that previous studies have already reported the emergence of non-albicans species as cause of candidemia [42–44] and considering our observation of a high frequency involvement of *C*. *parapsilosis* mostly in CCRBSI, we then focused on catheter-related BSI and on the species involved in this infection type. In the past years, several studies focused on BSI, highlighting the importance of biofilm production in the development of the intravascular devices–related candidemia [13, 24, 25]. *Candida* biofilm production has been described as a leading factor connected with the onset of a catheter-related candidemia and with the development of antifungal drug resistance [45–47]. Moreover, the role of biofilm in candidemia has been debated after the evidence that candidemia caused by biofilm-producing strains is characterized by worse outcome and mortality rates higher than BSIs caused by non-biofilm producing strains [2]. In our work, interestingly, analyzing the biofilm production capacity of the collected strains between 2011 and 2016, we observed a strong and significant estimated yearly increase of the number of biofilm producing strains. These data strongly indicated that the *Candida* population changed in our hospital during the six years considered, with a growing frequency of isolation of strains characterized by biofilm production capacity. We also observed that the species with the highest levels of intrinsic biofilm production was *C*. *albicans*, followed by *C*. *tropicalis*, thus confirming previous observations [25]. Intriguingly, we noticed a high number of biofilm producing strains not only for *C*. *albicans* but also for *C*. *parapsilosis* species (246 and 130 producer strains respectively), that were the most frequently isolated species of our collection. In particular, as we described above, we observed a higher number of *C*. *parapsilosis* isolation in the CCRBSI subgroup and statistical analyses reported a significant association between CCRBSI and being a producer strain (p = 0.0012). Although *C*. *parapsilosis* presented mean BI values lower than those of *C*. *albicans*, all these data pointed to a pivotal role for *C*. *parapsilosis* and its biofilm production capacity in the development of CCRBSI. These results are in line with those reported in our previous work [25], where we observed that intravascular device (IVD)-related BSIs were more often sustained by *C*. *parapsilosis*. It has been observed that *C*. *parapsilosis* needs glucose to form biofilm that might be found in the glucose-rich habitat, which is typical of parenteral nutrition [2]. Therefore, parenteral nutrition and administration of glucose-containing fluids through IVDs could promote biofilm formation by *C*. *parapsilosis* strains on the catheter surfaces, thus leading to the development of CCRBSI. At the same time, *C*. *parapsilosis* has been shown as the most frequently isolated species from the hands of hospital workers, thus increasing the risk of a contamination during the standard IVD clinical management [48–50]. In our work, this hypothesis seems to be confirmed by the higher incidence of *C*. *parapsilosis* isolation from the ICU, where IVDs are widely used [51]. To our knowledge, no previous studies have investigated the time trend of *Candida* biofilm production. From our data, a decrease in *C*. *parapsilosis* biofilm production in the ICU emerged during the time period considered, while no differences were observed in the medical and surgical wards. These data could be related to temporal variations of the consumption of antifungals acting on biofilm-producer strains, and that could have influenced the ecology of *Candida* species in our hospital. Indeed, PCA reflected with a very neat transition in year 2015. It was evident how the factor 1 (69% of total variance explained) was a ‘size’ component [33] accounting for the basic trend of relative proportion of antifungals use along the years. The second factor is on the contrary a ‘shape’ component (coexistence of positive and negative loadings), that identifies a very neat transition in year 2015 when it becomes prevalent (higher correlation) with respect to the ‘invariant profile’. In other words, in 2015 something happened provoking a ‘turning point’ in the antifungal drug usage profile. This phenomenon was explained by the construction of a ‘bi-dimensional’ confidence square in which the main factors involved were identified in the voriconazole use in the general medicine wards (Factor1), with almost constant administration during the first 4-year time period. The second temporal trend showed a correspondence to fluconazole use in the ICU, with a strong increase during the last two years. Concerning the latter, it was evident how the increased use of fluconazole, that was more than twenty-eight times higher than the mean use of years 2011–2013, was accompanied to a general increase in fluconazole MIC90 values, particularly for the MIC90 values of *C*. *parapsilosis*, in the ICU ward. These observations paralleled those of Lindberg et al, who recently showed reduced susceptibility of *C*. *parapsilosis* to fluconazole and echinocandins in a Swedish hospital [52]. Interestingly when considering the biofilm producer strains, the increase in fluconazole MIC90 values was registered not only for *C*. *parapsilosis* but also for *C*. *albicans*. Despite the emergence of fluconazole resistant *C*. *albicans* strains, mainly reported in vulvovaginal and oral mucosal infections [53, 54], and related to the wide use of this drug, the sensibility to this antifungal drug is stably at high levels, especially in BSI. To our knowledge no previous studies have reported an increase in fluconazole MIC90 for *C*. *albicans* involved in BSI. Further to fluconazole, we also observed that the consumption of amphotericin B and caspofungin in 2015 and 2016 was respectively seven and four times higher than the mean consumption observed in the years from 2011 to 2014. It could be, therefore, that the increase of the MIC50s of amphotericin B and caspofungin for both *C*. *albicans* and *C*. *parapsilosis* were related to an enhanced consumption of these antifungals. Of course, the real impact of this observation needs further evaluation, as the acquired resistance is uncommonly described [55]. Our study suffers of three major limitations. Firstly, being a retrospective analysis, mortality data and the antifungal treatment received by the single patient were not examined due to the difficulties in data recovering. Secondly, the DDDs data were also referred to the consumption of antifungals for the prophylaxis and therapy of infective episodes other than *Candida* BSI. Thirdly, the study lacks data regarding clonality of *Candida* isolates for implementing the changes of local microbiology over time.

## Conclusion

The increased number of *Candida* BSIs observed in our hospital has led both to an enhanced consumption of antifungals and to a marked increase of the MIC90 of fluconazole particularly in the ICU, as well as of the MIC50 for caspofungin and amphotericin B. Furthermore, a change in the ecology of the yeast population involved in BSI has been observed with an increased importance of *C*. *parapsilosis* that was the species more frequently involved in etiology of CCRBSI. In parallel, even though an increased cumulative trend of the number of biofilm producing strains has been observed, a decreased biofilm production by *C*. *parapsilosis* was observed in the ICU ward. Whether these observations are linked to a change in the drug consumption in a particular ward is a matter of debate, still the striking increase of fluconazole use in the ICU was accompanied to a change in the biofilm-producing population of the isolated yeasts. On a more general perspective, the increase in time of the biofilm producer strains (much faster than the non-producer one) can be considered as a potential threat to human health.
